# Metabolic score for insulin resistance is associated with metabolic dysfunction-associated steatotic liver disease among adults with hypertension: a cross-sectional secondary analysis of a Chinese single-center health check-up population

**DOI:** 10.3389/fendo.2026.1888652

**Published:** 2026-08-11

**Authors:** Feng Lu, Lei Qiu, Peng Yu, Tao Zhang, Guida Fang, Gang Wang, Yongchang Miao

**Affiliations:** 1 Department of Bariatric and Metabolic Surgery, The Second People’s Hospital of Lianyungang Affiliated to Kangda College of Nanjing Medical University, Lianyungang, Jiangsu, China; 2 Department of Gastrointestinal Surgery, The Second People’s Hospital of Lianyungang Affiliated to Kangda College of Nanjing Medical University, Lianyungang, Jiangsu, China

**Keywords:** health check-up population, hypertension, insulin resistance, MASLD, METS-IR, receiver operating characteristic curve, restricted cubic spline, secondary analysis

## Abstract

**Background:**

Metabolic dysfunction-associated steatotic liver disease (MASLD; formerly termed non-alcoholic fatty liver disease) and hypertension frequently coexist within the same cardiometabolic context. The metabolic score for insulin resistance (METS-IR), which is derived from body mass index, fasting blood glucose, triglycerides, and high-density lipoprotein cholesterol without requiring insulin measurement, may serve as a practical auxiliary indicator of metabolic dysfunction.

**Objective:**

This study investigated the association between METS-IR and MASLD among adults with hypertension and evaluated the dose-response pattern, non-linear features, discrimination, and effect modification.

**Methods:**

This secondary cross-sectional analysis used publicly available data from a Chinese single-center health check-up cohort. A total of 943 adults with hypertension were included. MASLD was operationally defined as ultrasonographically detected hepatic steatosis in adults with hypertension after exclusion of excessive alcohol intake and other known causes of liver disease. The primary multivariable model was adjusted for age group, sex, tobacco use, alcohol use, and diabetes; an extended model additionally included uric acid, total cholesterol, low-density lipoprotein cholesterol, and fat-to-muscle ratio. Logistic regression, quartile analysis, restricted cubic spline analysis, sensitivity analyses, receiver operating characteristic analysis, and subgroup interaction analyses were performed.

**Results:**

Among the 943 participants, 640 had MASLD, corresponding to a prevalence of 67.9%. The prevalence of MASLD increased across METS-IR quartiles from 40.7% to 67.2%, 74.6%, and 89.0%. After adjustment for age group, sex, tobacco use, alcohol use, and diabetes, each 1-standard deviation increase in METS-IR was associated with higher odds of prevalent MASLD (odds ratio [OR] = 2.78, 95% confidence interval [CI]: 2.24-3.44, P < 0.001). Compared with quartile 1, the ORs for quartiles 2, 3, and 4 were 2.99, 4.01, and 9.72, respectively. In the extended model, the corresponding OR per 1-standard deviation increase was 2.47 (95% CI: 1.91-3.18, P < 0.001). Restricted cubic spline analysis suggested mild non-linearity. Exploratory ROC analysis yielded an area under the curve of 0.738 (95% CI: 0.705-0.772); the data-derived METS-IR cut-point of 42.40 had a sensitivity of 53.0% and a specificity of 81.8%. A borderline interaction by sex was observed (P for interaction = 0.046), whereas interactions by age group and diabetes status were not statistically significant.

**Conclusion:**

Among adults with hypertension in a Chinese single-center health check-up population, higher METS-IR was associated with a higher likelihood of prevalent MASLD and showed a graded, mildly non-linear pattern. METS-IR may serve as an accessible auxiliary metabolic indicator, but its data-derived cut-point and possible sex-specific association require independent validation.

## Introduction

Metabolic dysfunction-associated steatotic liver disease (MASLD), formerly termed non-alcoholic fatty liver disease (NAFLD), is one of the most common chronic liver diseases worldwide. Its spectrum ranges from simple hepatic steatosis to metabolic dysfunction-associated steatohepatitis, liver fibrosis, cirrhosis, and hepatocellular carcinoma. The new nomenclature emphasizes hepatic steatosis in the presence of cardiometabolic risk factors (1). Systematic reviews and meta-analyses conducted under the former NAFLD terminology have shown that the disease represents an important global public health burden whose prevalence continues to increase alongside obesity and metabolic disorders (2, 3). Clinical management increasingly emphasizes comprehensive assessment of cardiometabolic risk rather than focusing only on the liver itself (4).

Hypertension and MASLD are closely linked. Evidence generated under the former NAFLD terminology has shown a strong association with cardiovascular disease, and the two conditions share mechanisms including insulin resistance, dyslipidemia, visceral adiposity, oxidative stress, and low-grade inflammation (5). Previous studies have suggested that hepatic steatosis may contribute to the development and progression of hypertension (6). Systematic reviews and meta-analyses have also reported a higher subsequent risk of hypertension and a potentially bidirectional relationship between the two conditions (7, 8). Therefore, identifying individuals with a high likelihood of MASLD among patients with hypertension has clinical relevance.

METS-IR is a surrogate index of insulin resistance based on routine health check-up variables, including body mass index (BMI), fasting blood glucose (FBG), triglycerides (TG), and high-density lipoprotein cholesterol (HDL-C). Compared with indices requiring insulin measurement, METS-IR is easier to obtain in health examination centers, primary care, and chronic disease follow-up. METS-IR was initially shown to reflect insulin sensitivity and to predict visceral adiposity and incident type 2 diabetes (9). Subsequent studies conducted under the former NAFLD terminology suggested associations with incident hepatic steatosis, disease in non-obese populations, and blood-pressure-related metabolic outcomes (10–12). However, existing studies have mainly focused on general populations, non-obese individuals, or other metabolic populations. Whether METS-IR can identify MASLD-related metabolic burden among adults who already have hypertension remains insufficiently characterized.

Therefore, using publicly available single-center health check-up data, this study evaluated the association between METS-IR and prevalent MASLD among adults with hypertension. We further examined the dose-response pattern, potential non-linearity, discrimination, robustness, and effect modification by sex, age group, and diabetes status.

## Materials and methods

### Data source and study population

This study was a secondary cross-sectional analysis of publicly available data. The data were obtained from the Dryad dataset entitled “Association of fat-to-muscle ratio with non-alcoholic fatty liver disease: a single-center retrospective study” (DOI: 10.5061/dryad.7d7wm3809). The dataset originated from a Chinese single-center retrospective study of health check-up participants published by Yan et al. (13). The original study consecutively enrolled 1,830 participants aged 40–79 years who underwent health examinations, body composition analysis, and liver ultrasonography at Wuhan Union Hospital between January 2020 and November 2021. The age range of 40–79 years was inherited from the eligibility criteria of the source cohort rather than selected specifically for the present secondary analysis. After excluding participants with excessive alcohol intake, viral liver disease, autoimmune liver disease, drug-induced liver disease, other specified liver diseases, acute disease, renal insufficiency, active malignancy, oral or injectable glucocorticoid use, or missing key biochemical or medical history data, 1,592 participants were included in the original analysis. The dataset contains demographic characteristics, lifestyle factors, body composition indicators, blood pressure, biochemical indicators, hypertension, diabetes, and the original binary NAFLD variable. In the present analysis, we selected 943 participants who met the source-study definition of hypertension. There were no missing values in the variables used in the hypertensive subsample; therefore, no imputation was performed.

The original study was approved by the Ethics Committee of Tongji Medical College, Huazhong University of Science and Technology (S155). Because the original study used anonymous retrospective medical data, the requirement for informed consent was waived. The present study used publicly available de-identified data for secondary analysis, did not contact participants, and did not obtain any personally identifiable information. Ethical approval and informed consent requirements were therefore based on the original study.

### Exposure variable

The primary exposure was METS-IR. Because FBG, TG, and HDL-C in the original dataset were recorded in mmol/L, they were converted to mg/dL before calculation using standard conversion factors: FBG x 18, TG x 88.57, and HDL-C x 38.67. METS-IR was calculated according to the formula proposed by Bello-Chavolla et al. (9): METS-IR = {ln[(2 x FBG [mg/dL]) + TG (mg/dL)] x BMI (kg/m2)}/ln[HDL-C (mg/dL)], where ln denotes the natural logarithm. In the analyses, METS-IR was treated as a continuous variable, per 1-standard deviation increase, and as a quartile-based categorical variable. The quartile cut-points were 36.78, 40.98, and 46.22.

### Outcome variable and covariates

The source dataset coded the outcome as NAFLD because it was established before adoption of the current steatotic liver disease nomenclature. In the present study, the outcome was operationally reclassified as MASLD. Fatty liver was identified by routine abdominal B-mode ultrasonography. According to the source study, participants with excessive alcohol intake (>210 g/week in men and >140 g/week in women), viral liver disease, autoimmune liver disease, drug-induced liver disease, or other specified liver diseases had been excluded (13). Because all participants included in the present analysis were classified as having hypertension, they fulfilled a cardiometabolic risk criterion. This operational reclassification was based on variables available in the public dataset and did not constitute an independent clinical reassessment using complete patient-level information. The public dataset did not indicate whether ultrasonographically detected hepatic steatosis represented a first diagnosis or a previously recognized condition.

Covariates were selected based on previous literature, clinical relevance, and data availability, and included age group, sex, tobacco use, alcohol use, diabetes, uric acid (UA), total cholesterol (TC), low-density lipoprotein cholesterol (LDL-C), fat-to-muscle ratio (FMR), systolic blood pressure (SBP), and diastolic blood pressure (DBP). Hypertension was defined according to the source study as a self-reported history of hypertension or current use of oral antihypertensive medication. Blood pressure measurements obtained during the health examination were not used to newly classify hypertension in the present secondary analysis. Diabetes was defined as a self-reported history of diabetes or current use of glucose-lowering medication; FBG or HbA1c criteria were not used to newly classify diabetes. Age was recorded as a categorical variable, with codes 1, 2, 3, and 4 corresponding to 40-49, 50-59, 60-69, and 70–79 years, respectively, and was treated as categorical in regression models. Because the METS-IR formula includes BMI, FBG, TG, and HDL-C, these component variables were not included as covariates in the main regression models to avoid overadjustment and unnecessary model overlap. UA, TC, LDL-C, and FMR were included in the extended model to examine robustness, although these variables may lie on complex metabolic pathways and the corresponding results were interpreted as supplementary. Alanine aminotransferase (ALT) and aspartate aminotransferase (AST) may reflect MASLD-related hepatocellular injury and were therefore not included as main-model covariates, but were used in sensitivity analyses.

### Statistical analysis

Continuous variables are presented as mean +/- standard deviation or median (interquartile range), according to distributional characteristics, and categorical variables are presented as n (%). In the baseline table, ALT, AST, TC, TG, and FBG were treated as non-normally distributed variables and compared using the Mann-Whitney U test. Other continuous variables were compared using Student’s t-test. Categorical variables were compared using Pearson’s chi-square test without continuity correction.

Logistic regression was used to estimate odds ratios (ORs) and 95% confidence intervals (CIs) for the association between METS-IR and prevalent MASLD. The crude model was unadjusted. Model 1 was adjusted for age group and sex. Model 2 was further adjusted for tobacco use, alcohol use, and diabetes and was used as the primary interpretive model. Model 3 was additionally adjusted for UA, TC, LDL-C, and FMR on the basis of Model 2 and was used as an extended adjustment model. Because this was a cross-sectional study and the prevalence of MASLD was high, ORs were used to represent relative differences in the odds of prevalent MASLD and were not interpreted as relative risks or prevalence ratios.

Restricted cubic spline (RCS) analysis was used to evaluate the potential non-linear association between METS-IR and MASLD. The RCS model was adjusted for the same variables as Model 2. Four knots were placed at the 5th, 35th, 65th, and 95th percentiles of METS-IR, and the reference point was set at the median METS-IR value of 40.98. The RCS curve was displayed within the 1st to 99th percentile range of METS-IR to reduce the influence of sparse extreme values. Exploratory breakpoint analysis was performed within the same range to describe possible changes in slope. This analysis was interpreted as exploratory and was not considered a clinical diagnostic, screening, or intervention threshold.

To examine robustness, several sensitivity analyses were performed. First, SBP and DBP were further adjusted. Second, ALT and AST were added to Model 3; because these enzymes may be affected by MASLD, this analysis was interpreted as exploratory. Third, participants with diabetes were excluded. Finally, the analysis was restricted to participants within the 1st to 99th percentile range of METS-IR to assess the influence of extreme values.

Exploratory receiver operating characteristic (ROC) curve analysis was performed to assess the discrimination of METS-IR for prevalent MASLD. The area under the curve (AUC) and its 95% CI were estimated, and the Youden index, defined as sensitivity plus specificity minus one, was used to identify a data-derived cut-point. Because the cut-point was derived and evaluated in the same single-center sample, it was considered exploratory and was not interpreted as a validated clinical screening or diagnostic threshold.

Prespecified subgroup analyses were conducted according to sex, age group, and diabetes status. METS-IR was standardized using the standard deviation of the overall hypertensive sample. Sex-stratified models were adjusted for age group, tobacco use, alcohol use, and diabetes; age-stratified models were adjusted for sex, tobacco use, alcohol use, and diabetes; and diabetes-stratified models were adjusted for age group, sex, tobacco use, and alcohol use. P values for interaction were obtained using likelihood ratio tests comparing models with and without the corresponding multiplicative interaction terms. For categorical age group, the three interaction terms were tested jointly using a 3-degree-of-freedom likelihood ratio test. All tests were two-sided, and P < 0.05 was considered statistically significant.

## Results

### Study population and baseline characteristics

Among the 1,592 participants in the original dataset, 943 had hypertension, of whom 640 had MASLD. The prevalence of MASLD was 67.9%. Compared with participants without MASLD, those with MASLD had higher proportions of men and diabetes, higher levels of BMI, METS-IR, ALT, AST, UA, FBG, TC, TG, and LDL-C, and lower levels of HDL-C. SBP levels were similar between the two groups, whereas DBP was slightly higher in the MASLD group but did not reach statistical significance. Baseline characteristics are shown in **Table 1**.

**Table 1:** Baseline characteristics of hypertensive participants according to MASLD status.

Variable	Total (n=943)	Non-MASLD (n=303)	MASLD (n=640)	P value
Age group, years				<0.001
40-49	142 (15.1)	28 (9.2)	114 (17.8)	
50-59	393 (41.7)	115 (38.0)	278 (43.4)	
60-69	273 (29.0)	104 (34.3)	169 (26.4)	
70-79	135 (14.3)	56 (18.5)	79 (12.3)	
Male	740 (78.5)	225 (74.3)	515 (80.5)	0.030
Tobacco use	361 (38.3)	103 (34.0)	258 (40.3)	0.062
Alcohol use	344 (36.5)	99 (32.7)	245 (38.3)	0.095
Diabetes	379 (40.2)	83 (27.4)	296 (46.2)	<0.001
BMI, kg/m²	26.2 ± 2.9	24.9 ± 2.4	26.8 ± 2.9	<0.001
METS-IR	41.9 ± 7.3	38.0 ± 5.8	43.8 ± 7.2	<0.001
FMR	0.41 ± 0.12	0.39 ± 0.12	0.41 ± 0.12	0.005
PLT, 10^9/L	209.7 ± 54.1	206.6 ± 52.3	211.2 ± 55.0	0.224
ALT, U/L	23.0 (16.0, 32.0)	19.0 (14.0, 26.0)	25.0 (18.0, 35.0)	<0.001
AST, U/L	21.0 (18.0, 27.0)	20.0 (17.0, 24.0)	22.0 (18.0, 28.0)	<0.001
UA, μmol/L	381.2 ± 93.7	362.9 ± 83.6	389.8 ± 97.0	<0.001
FBG, mmol/L	5.3 (4.8, 6.0)	5.0 (4.6, 5.5)	5.4 (4.9, 6.3)	<0.001
TC, mmol/L	4.2 (3.5, 5.0)	4.1 (3.3, 4.8)	4.3 (3.6, 5.1)	<0.001
TG, mmol/L	1.5 (1.0, 2.3)	1.1 (0.8, 1.6)	1.8 (1.2, 2.6)	<0.001
HDL-C, mmol/L	1.1 ± 0.3	1.1 ± 0.3	1.0 ± 0.3	<0.001
LDL-C, mmol/L	2.5 ± 0.9	2.5 ± 0.9	2.6 ± 0.9	0.042
SBP, mmHg	135.8 ± 15.9	135.9 ± 14.8	135.8 ± 16.3	0.891
DBP, mmHg	84.4 ± 11.5	83.4 ± 11.1	84.9 ± 11.6	0.079

Values are shown as n (%), mean ± SD, or median (IQR). ALT, AST, TC, TG, and FBG were summarized as median (IQR) and compared by the Mann-Whitney U test. Other continuous variables were compared by Student t test. Categorical variables were compared by Pearson chi-square test without continuity correction. ALT, alanine aminotransferase; AST, aspartate aminotransferase; BMI, body mass index; DBP, diastolic blood pressure; FBG, fasting blood glucose; FMR, fat-to-muscle ratio; HDL-C, high-density lipoprotein cholesterol; LDL-C, low-density lipoprotein cholesterol; METS-IR, metabolic score for insulin resistance; MASLD, metabolic dysfunction-associated steatotic liver disease; PLT, platelet count; SBP, systolic blood pressure; TC, total cholesterol; TG, triglycerides; UA, uric acid.

When participants were grouped by METS-IR quartiles, the proportions of men, tobacco users, alcohol users, participants with diabetes, and participants with MASLD increased with higher METS-IR, as did BMI, TG, and ALT levels, whereas HDL-C levels decreased. Baseline characteristics according to METS-IR quartiles are shown in **Supplementary Table 1**.

### METS-IR quartiles and MASLD

**Figure 1:**
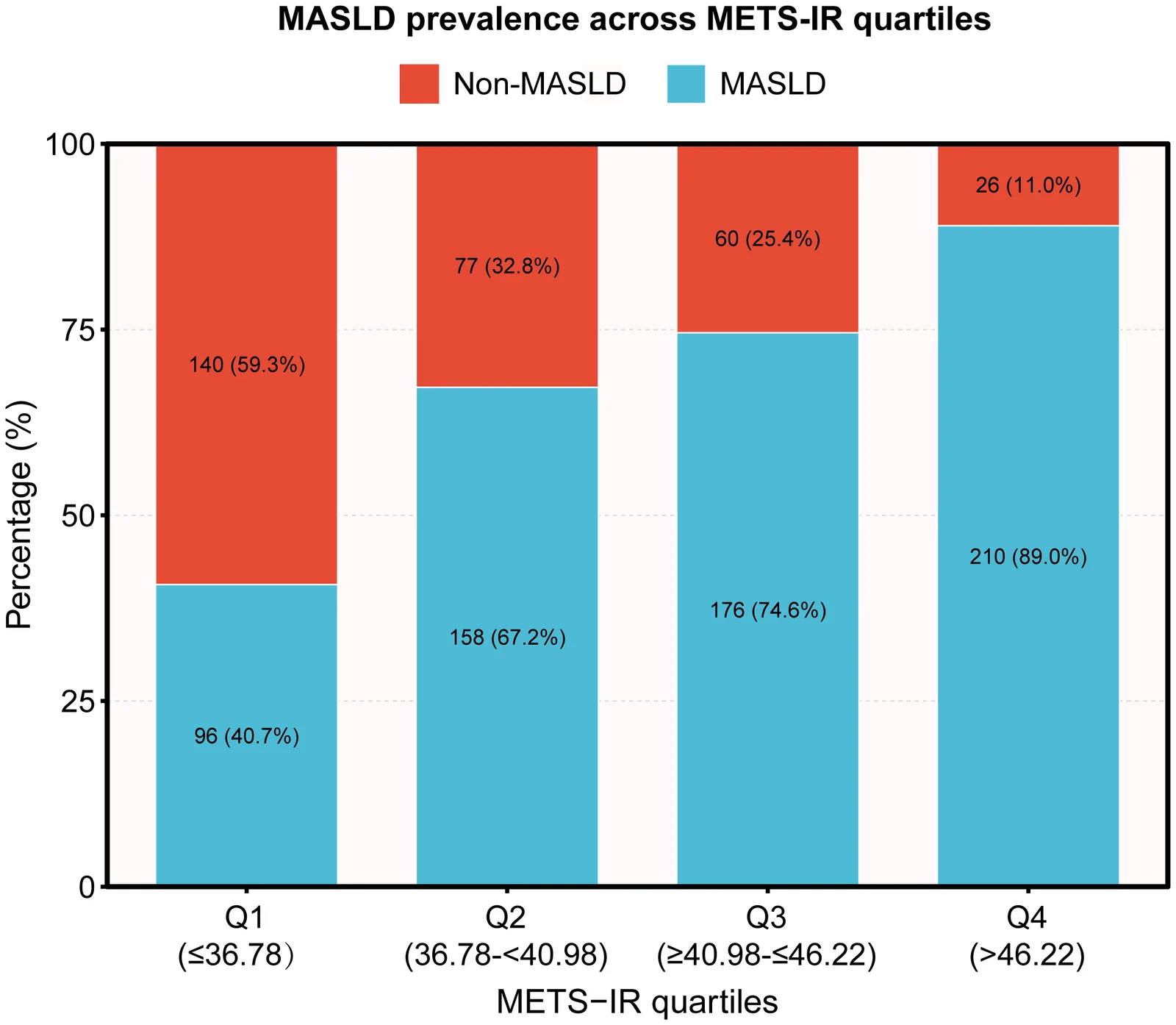
Prevalence of MASLD across METS-IR quartiles among adults with hypertension. The quartile cut-points were Q1 <=36.78, Q2 >36.78 to <40.98, Q3 >=40.98 to <=46.22, and Q4 >46.22. The numbers of participants with MASLD were 96/236, 158/235, 176/236, and 210/236, corresponding to prevalences of 40.7%, 67.2%, 74.6%, and 89.0%, respectively. Alt text: Stacked bar chart showing a progressive increase in MASLD prevalence across METS-IR quartiles from 40.7% in Q1 to 89.0% in Q4.

The mean METS-IR value among adults with hypertension was 41.9 +/- 7.3. After grouping by quartiles, the prevalence of MASLD increased in a graded manner from 40.7% in quartile 1 to 67.2%, 74.6%, and 89.0% in quartiles 2, 3, and 4, respectively (**Figure 1**).

Logistic regression showed that METS-IR was significantly associated with prevalent MASLD whether modeled as a continuous variable, per 1-standard deviation increase, or as quartile categories (**Table 2**). In Model 2, each 1-unit increase in METS-IR was associated with an approximately 15% increase in the odds of prevalent MASLD (OR = 1.15, 95% CI: 1.12-1.18, P < 0.001). Each 1-standard deviation increase was associated with an approximately 178% increase in the odds of prevalent MASLD (OR = 2.78, 95% CI: 2.24-3.44, P < 0.001). Compared with quartile 1, the ORs were 2.99 (95% CI: 2.03-4.41), 4.01 (95% CI: 2.67-6.05), and 9.72 (95% CI: 5.84-16.17) for quartiles 2, 3, and 4, respectively (all P < 0.001). The trend test was significant.

**Table 2:** Association between METS-IR and MASLD among adults with hypertension.

Exposure	n	Events	Crude OR (95% CI) P value	Model 1 OR (95% CI) P value	Model 2 OR (95% CI) P value	Model 3 OR (95% CI) P value
METS-IR per 1-unit increase	943	640	1.16 (1.13-1.19) <0.001	1.16 (1.13-1.19) <0.001	1.15 (1.12-1.18) <0.001	1.13 (1.09-1.17) <0.001
METS-IR per 1-SD increase	943	640	2.94 (2.41-3.60) <0.001	2.97 (2.41-3.66) <0.001	2.78 (2.24-3.44) <0.001	2.47 (1.91-3.18) <0.001
Q1	236	96	1.00 (Reference)	1.00 (Reference)	1.00 (Reference)	1.00 (Reference)
Q2	235	158	2.99 (2.05-4.36) <0.001	3.15 (2.15-4.64) <0.001	2.99 (2.03-4.41) <0.001	2.85 (1.90-4.30) <0.001
Q3	236	176	4.28 (2.89-6.33) <0.001	4.34 (2.90-6.49) <0.001	4.01 (2.67-6.05) <0.001	3.34 (2.11-5.28) <0.001
Q4	236	210	11.78 (7.27-19.10) <0.001	11.56 (7.02-19.02) <0.001	9.72 (5.84-16.17) <0.001	6.70 (3.75-11.97) <0.001
P for trend	–	–	<0.001	<0.001	<0.001	<0.001

Crude model: unadjusted. Model 1: adjusted for age group and sex. Model 2: adjusted for age group, sex, tobacco use, alcohol use, and diabetes. Model 3: adjusted for variables in Model 2 plus UA, TC, LDL-C, and FMR. Q1 was used as the reference category. Quartile cut-offs were rounded to two decimal places: Q1 ≤36.78; Q2 >36.78 to <40.98; Q3 ≥40.98 to ≤46.22; Q4 >46.22. CI, confidence interval; FMR, fat-to-muscle ratio; LDL-C, low-density lipoprotein cholesterol; METS-IR, metabolic score for insulin resistance; MASLD, metabolic dysfunction-associated steatotic liver disease; OR, odds ratio; TC, total cholesterol; UA, uric acid.

In the extended adjustment model (Model 3), each 1-standard deviation increase in METS-IR remained associated with higher odds of prevalent MASLD (OR = 2.47, 95% CI: 1.91-3.18, P < 0.001). Compared with quartile 1, the ORs for quartiles 2, 3, and 4 were 2.85, 3.34, and 6.70, respectively. Quartile-based results from additional sensitivity models are shown in **Supplementary Table 2**.

### Non-linearity and exploratory breakpoint analysis

**Figure 2:**
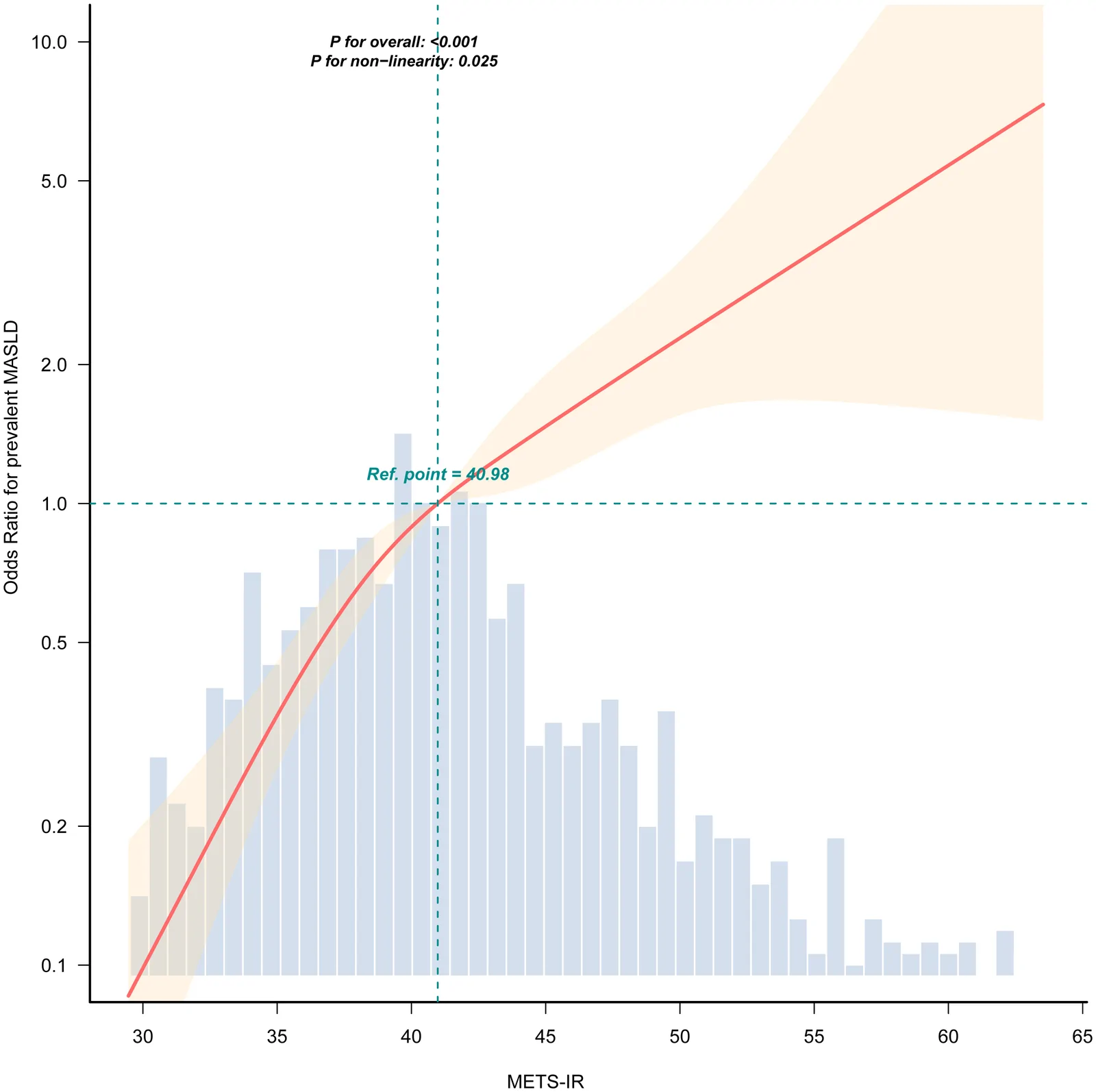
Restricted cubic spline association between METS-IR and prevalent MASLD among adults with hypertension. The logistic regression model was adjusted for age group, sex, tobacco use, alcohol use, and diabetes. Four knots were placed at the 5th, 35th, 65th, and 95th percentiles of METS-IR, and the median value of 40.98 was used as the reference. The solid line represents the OR, the shaded area represents the 95% CI, and the histogram shows the distribution of METS-IR. The curve was displayed from the 1st to the 99th percentile. P for overall association <0.001 and P for non-linearity =0.025. Alt text: Restricted cubic spline plot showing increasing odds of prevalent MASLD with higher METS-IR and mild non-linearity.

RCS analysis showed that, after adjustment for age group, sex, tobacco use, alcohol use, and diabetes, METS-IR was positively associated with MASLD overall (P for overall < 0.001), with evidence of mild non-linearity (P for non-linearity = 0.025) (**Figure 2**). Using the median METS-IR value of 40.98 as the reference point, the odds of prevalent MASLD increased with higher METS-IR.

Exploratory breakpoint analysis was performed within the 1st to 99th percentile range of METS-IR and included 923 participants, of whom 628 had MASLD. The estimated breakpoint was 38.74 (95% CI: 38.61-38.87). When METS-IR was <=38.74, each 1-unit increase was associated with an approximately 29% increase in the odds of prevalent MASLD (OR = 1.29, 95% CI: 1.16-1.43, P < 0.001). When METS-IR was >38.74, each 1-unit increase remained associated with an approximately 11% increase in the odds of prevalent MASLD (OR = 1.11, 95% CI: 1.06-1.17, P < 0.001). The likelihood ratio test yielded P = 0.006. Results are shown in **Supplementary Table 3**; the estimated breakpoint was not treated as a clinical threshold.

### Sensitivity analyses

The sensitivity analyses were consistent with the main findings (**Table 3**). For each 1-standard deviation increase in METS-IR, the OR in Model 2 was 2.78 (95% CI: 2.24-3.44, P < 0.001). The estimate was unchanged after further adjustment for SBP and DBP. After additionally including ALT and AST on the basis of Model 3, the association remained statistically significant. The findings also remained stable after excluding participants with diabetes and after restricting the analysis to the 1st to 99th percentile range of METS-IR.

**Table 3:** Sensitivity analyses for the association between METS-IR and MASLD.

Analysis	n	Events	OR per 1-SD increase in METS-IR (95% CI)	P value
Main adjusted model (Model 2)	943	640	2.78 (2.24-3.44)	<0.001
Additionally adjusted for SBP and DBP	943	640	2.78 (2.24-3.44)	<0.001
Additionally adjusted for Model 3 covariates plus ALT and AST	943	640	2.32 (1.79-3.01)	<0.001
Excluding participants with diabetes	564	344	3.25 (2.45-4.30)	<0.001
Restricting to the 1st-99th percentile range of METS-IR*	923	628	2.74 (2.20-3.42)	<0.001

Unless otherwise indicated, estimates are ORs per 1-SD increase in METS-IR. The main adjusted model was Model 2, adjusted for age group, sex, tobacco use, alcohol use, and diabetes. Model 3 covariates included UA, TC, LDL-C, and FMR. *This analysis excluded participants with METS-IR values below the 1st percentile or above the 99th percentile; 923 participants were included. ALT, alanine aminotransferase; AST, aspartate aminotransferase; CI, confidence interval; DBP, diastolic blood pressure; FMR, fat-to-muscle ratio; LDL-C, low-density lipoprotein cholesterol; METS-IR, metabolic score for insulin resistance; MASLD, metabolic dysfunction-associated steatotic liver disease; OR, odds ratio; SBP, systolic blood pressure; TC, total cholesterol; UA, uric acid.

These sensitivity analyses suggest that the association between METS-IR and MASLD was not entirely driven by blood pressure level, diabetes status, liver enzyme differences, or extreme METS-IR values.

### Exploratory ROC analysis

Exploratory ROC analysis showed that METS-IR had moderate discrimination for prevalent MASLD, with an AUC of 0.738 (95% CI: 0.705-0.772) (**Supplementary Figure 1**). The maximum Youden index was 0.348, corresponding to an original-scale METS-IR cut-point of 42.40 and a univariable model-predicted probability of 0.734, with a sensitivity of 53.0% and a specificity of 81.8%. Because the cut-point was derived and evaluated in the same sample, it was considered exploratory rather than a validated clinical threshold.

### Subgroup and interaction analyses

The positive association between METS-IR and prevalent MASLD was observed across sex, age-group, and diabetes subgroups (**Supplementary Table 4**). The adjusted OR per 1-standard deviation increase in METS-IR was 5.29 (95% CI: 2.91-9.64) in women and 2.48 (95% CI: 1.97-3.13) in men. A borderline interaction by sex was observed (P for interaction = 0.046). No statistically significant interactions were detected for age group or diabetes status (P for interaction = 0.533 and 0.220, respectively).

## Discussion

The prevalence of MASLD among adults with hypertension was 67.9%, and METS-IR showed a strong graded association with prevalent MASLD. The association remained significant after adjustment for demographic characteristics, lifestyle factors, diabetes, conventional metabolic variables, and fat-to-muscle ratio. RCS analysis indicated mild non-linearity, and sensitivity analyses yielded consistent results. Exploratory analyses showed moderate discrimination and suggested possible effect modification by sex. These findings demonstrate an association but do not establish causality, diagnostic superiority, or a validated screening threshold.

The present findings provide evidence for a new clinical context based on previous studies of METS-IR. Prior studies have shown a non-linear dose-response association between METS-IR and MASLD and a significant association between METS-IR and MASLD among patients with type 2 diabetes (14, 15). Regarding blood pressure abnormalities, METS-IR has been reported to be associated with prevalent and incident hypertension (16, 17). METS-IR has also been associated with ischemic heart disease, all-cause mortality, and cardiovascular mortality (18, 19). These studies suggest that METS-IR may reflect not only a single liver-related metabolic abnormality but also a broader cardiometabolic burden. The incremental value of the present study lies in evaluating METS-IR among individuals who already have hypertension. Patients with hypertension already have elevated cardiometabolic risk, and clinical follow-up often focuses on blood pressure control and prevention of vascular events, whereas hepatic fat accumulation may be underrecognized. This study found that METS-IR remained significantly associated with prevalent MASLD in this specific population and showed a dose-response pattern that was robust to further adjustment for multiple metabolic indicators and body composition variables. These findings suggest that METS-IR may be a low-cost auxiliary marker for identifying possible MASLD in hypertension management and health check-up settings.

The association between METS-IR and MASLD is biologically plausible. METS-IR consists of BMI, FBG, TG, and HDL-C, which reflect body weight burden, glucose metabolic status, triglyceride enrichment, and protective lipoprotein levels, respectively. Insulin resistance can promote lipolysis in adipose tissue, increase the delivery of free fatty acids to the liver, and enhance hepatic *de novo* lipogenesis. At the same time, imbalance in very-low-density lipoprotein export, oxidative stress, mitochondrial dysfunction, and inflammatory responses may jointly promote hepatic fat accumulation and hepatocellular injury (20). Metabolic risk factors are also associated with MASLD progression and risk of severe liver disease, indicating that hepatic fat accumulation is often part of systemic metabolic dysfunction (21). The association between MASLD and risk of diabetes further supports the central role of insulin resistance in hepatic and systemic metabolic abnormalities (22). In the context of hypertension, insulin resistance is also related to sympathetic activation, renin-angiotensin system activation, sodium retention, and endothelial dysfunction, processes that may reinforce the interaction between hepatic fat accumulation and vascular-metabolic abnormalities.

The RCS and breakpoint analyses suggested that the association between METS-IR and MASLD was not completely linear. Overall, higher METS-IR corresponded to higher odds of prevalent MASLD. However, the segmented results suggested that the marginal increase differed across METS-IR ranges. The estimated breakpoint was approximately 38.74. Below this value, each 1-unit increase in METS-IR was associated with an approximately 29% increase in the odds of prevalent MASLD. Above this value, each 1-unit increase in METS-IR remained associated with an approximately 11% increase in the odds of prevalent MASLD, indicating that the association continued to rise but with a relatively lower slope. This pattern may reflect the rapid accumulation of hepatic steatosis-related phenotypes during the transition from lower to intermediate levels of metabolic dysfunction. At higher METS-IR levels, many individuals may already have obesity, dysglycemia, dyslipidemia, or diabetes, and the probability of MASLD may already be high; therefore, the marginal increase in odds corresponding to each additional METS-IR unit may be smaller. It should be emphasized that the breakpoint identified in this study was an exploratory statistical finding. It cannot replace imaging-based diagnosis and should not be used as a clinical diagnostic, screening, or intervention threshold.

This study has several clinical implications. Routine management of patients with hypertension generally focuses on blood pressure control, target-organ damage, and cardiovascular event prevention. MASLD, as an important hepatic manifestation of systemic metabolic dysfunction, may be insufficiently recognized during routine follow-up. Studies have shown that the burden of MASLD and advanced fibrosis is clinically meaningful across blood pressure categories (23). Studies on the directionality of the relationship between MASLD and hypertension have also suggested that the two conditions may have mutually reinforcing metabolic links (24, 25). In the present study, the prevalence of MASLD among adults with hypertension reached 67.9%, indicating a high liver metabolic burden in this population. The advantage of METS-IR is that it is entirely based on routine health check-up indicators, including BMI, FBG, TG, and HDL-C, and does not require insulin measurement or additional laboratory costs. Therefore, METS-IR may serve as an opportunistic indicator in health check-up or follow-up settings for adults with hypertension, helping clinicians identify individuals who may require further liver ultrasonography, non-invasive assessment of liver fibrosis, or intensified metabolic intervention. However, METS-IR cannot replace imaging or clinical diagnosis. In the absence of prospective validation and prediction-performance evaluation, its more appropriate role is as a low-cost and easily accessible auxiliary identification tool.

A major feature of this study is its focus on hypertension, a cardiometabolic high-risk condition, and the evaluation of METS-IR in the context of hypertension management and health check-up screening. Previous studies based on the same public health check-up dataset have examined body composition, uric-acid-related indicators, and MASLD prediction models from different perspectives, including the associations of FMR with hypertension and uric acid with MASLD among patients with hypertension (26, 27). The same dataset has also been used in secondary studies of the uric acid-to-HDL-C ratio and MASLD and in MASLD prediction models (28–30). Compared with these studies, the present study focuses on the association between METS-IR, an index based on routine health check-up indicators, and prevalent MASLD among adults with hypertension, providing a different clinical perspective. In addition, this study combined conventional logistic regression with quartile-based dose-response analysis, RCS analysis, exploratory breakpoint analysis, and multiple sensitivity analyses. Thus, it not only described whether METS-IR was associated with MASLD but also characterized how the association varied across the METS-IR distribution.

Exploratory ROC analysis indicated moderate discrimination, with an AUC of 0.738. The data-derived cut-point of 42.40 showed relatively high specificity but limited sensitivity. METS-IR may therefore be more appropriate as an auxiliary metabolic indicator than as a stand-alone screening or exclusion test. Because the cut-point was derived and assessed in the same single-center sample, external validation is required before clinical use.

The association was numerically stronger in women than in men, and the likelihood ratio test yielded a borderline P value for interaction. However, this subgroup analysis was exploratory, the female subgroup was substantially smaller than the male subgroup, and multiple subgroup comparisons were conducted. The possible sex difference should therefore be interpreted cautiously and confirmed in larger independent studies.

This study also has several limitations. First, the cross-sectional design precludes causal inference and does not establish whether elevated METS-IR precedes MASLD. Second, the outcome was operationally reclassified from the binary NAFLD variable in the source dataset and was based mainly on abdominal ultrasonography. The available data did not capture steatosis grade, metabolic dysfunction-associated steatohepatitis, or fibrosis stage; therefore, the association between METS-IR and disease severity could not be evaluated. Third, the single-center health check-up setting substantially limits external validity. The age range of 40–79 years was inherited from the source cohort, limiting generalizability to younger adults and individuals aged 80 years or older. Fourth, detailed information on antihypertensive agents, lipid-lowering therapy, glucose-lowering drug classes, medication doses, treatment duration, and adherence was unavailable; medication-related residual confounding could not be excluded. Information on waist circumference, insulin, homeostatic model assessment of insulin resistance, diet, physical activity, and inflammatory markers was also unavailable. Fifth, METS-IR comprises BMI, FBG, TG, and HDL-C, which are closely related to the metabolic phenotype of MASLD; the contribution of the composite index cannot be fully separated from that of its components. Sixth, because MASLD prevalence was high, ORs should not be interpreted as relative risks. Seventh, the ROC cut-point was derived and evaluated in the same sample and may be optimistic; it should not be regarded as a validated clinical threshold. Finally, the subgroup and interaction analyses were exploratory and involved multiple comparisons without formal multiplicity adjustment, so the borderline sex interaction requires independent confirmation. Future prospective multicenter studies are needed to validate the association, discrimination, and clinical application boundaries of METS-IR among adults with hypertension.

## Conclusion

Among adults with hypertension in a Chinese single-center health check-up population, METS-IR was positively associated with prevalent MASLD and showed a graded, mildly non-linear pattern. Exploratory ROC analysis indicated moderate discrimination, while subgroup analyses suggested a possible sex difference that requires confirmation. METS-IR may serve as an accessible auxiliary metabolic indicator, but it cannot replace imaging-based diagnosis, and its data-derived cut-point requires prospective external validation.

## Data Availability

The original contributions presented in the study are included in the article/**Supplementary Material**. Further inquiries can be directed to the corresponding authors.

## Supplementary material

Supplementary Figure 1 Receiver operating characteristic curve of METS-IR for identifying prevalent MASLD among adults with hypertension. The AUC was 0.738 (95% CI: 0.705-0.772). The point annotated on the curve represents the univariable model-predicted probability cut-point of 0.734 (specificity, 0.818; sensitivity, 0.530), corresponding to an original-scale METS-IR cut-point of 42.40. The maximum Youden index was 0.348. Alt text: Receiver operating characteristic curve showing moderate discrimination of METS-IR for prevalent MASLD, with an AUC of 0.738.
