# Supplementary material for: Metabolic score for insulin resistance is associated with metabolic dysfunction-associated steatotic liver disease among adults with hypertension: a cross-sectional secondary analysis of a Chinese single-center health check-up population
Source: Front Endocrinol (Lausanne). 2026 Aug 11;17:1888652. doi: 10.3389/fendo.2026.1888652 (PMC13503243; doi:10.3389/fendo.2026.1888652)
Supplement: Supplementary file 1 [file SupplementaryFile1.docx]

**Supplementary Tables**

**Supplementary Table 1. Baseline characteristics according to METS-IR quartiles**

| **Variable** | **Q1 (≤36.78)** | **Q2 (>36.78-<40.98)** | **Q3 (≥40.98-≤46.22)** | **Q4 (>46.22)** | **P value** |
| --- | --- | --- | --- | --- | --- |
| Age group, years |  |  |  |  | <0.001 |
| 40-49 | 27 (11.4) | 23 (9.8) | 38 (16.1) | 54 (22.9) |  |
| 50-59 | 80 (33.9) | 95 (40.4) | 105 (44.5) | 113 (47.9) |  |
| 60-69 | 88 (37.3) | 70 (29.8) | 65 (27.5) | 50 (21.2) |  |
| 70-79 | 41 (17.4) | 47 (20.0) | 28 (11.9) | 19 (8.1) |  |
| Male | 147 (62.3) | 181 (77.0) | 199 (84.3) | 213 (90.3) | <0.001 |
| Tobacco use | 59 (25.0) | 85 (36.2) | 100 (42.4) | 117 (49.6) | <0.001 |
| Alcohol use | 58 (24.6) | 83 (35.3) | 101 (42.8) | 102 (43.2) | <0.001 |
| Diabetes | 58 (24.6) | 87 (37.0) | 97 (41.1) | 137 (58.1) | <0.001 |
| MASLD | 96 (40.7) | 158 (67.2) | 176 (74.6) | 210 (89.0) | <0.001 |
| BMI, kg/m² | 23.6 ± 1.4 | 25.2 ± 1.5 | 26.6 ± 1.8 | 29.3 ± 3.0 | <0.001 |
| METS-IR | 33.7 ± 2.1 | 39.0 ± 1.2 | 43.2 ± 1.4 | 51.9 ± 5.3 | <0.001 |
| FMR | 0.38 ± 0.12 | 0.40 ± 0.12 | 0.41 ± 0.11 | 0.44 ± 0.12 | <0.001 |
| PLT, 10^9/L | 207.7 ± 53.5 | 210.9 ± 53.3 | 210.1 ± 56.9 | 210.2 ± 53.2 | 0.927 |
| ALT, U/L | 19.0 (14.0, 26.0) | 23.0 (16.0, 30.5) | 23.0 (17.0, 33.2) | 29.0 (21.0, 40.0) | <0.001 |
| AST, U/L | 21.0 (17.0, 24.0) | 21.0 (17.0, 26.0) | 21.0 (17.0, 27.0) | 23.0 (19.0, 29.2) | <0.001 |
| UA, μmol/L | 352.6 ± 83.6 | 369.1 ± 89.5 | 396.7 ± 88.9 | 406.2 ± 102.4 | <0.001 |
| FBG, mmol/L | 4.9 (4.6, 5.4) | 5.1 (4.6, 5.7) | 5.4 (4.8, 6.1) | 5.7 (5.1, 7.0) | <0.001 |
| TC, mmol/L | 4.3 (3.5, 5.1) | 4.1 (3.5, 4.9) | 4.3 (3.4, 5.0) | 4.3 (3.7, 5.0) | 0.301 |
| TG, mmol/L | 1.0 (0.8, 1.3) | 1.3 (1.0, 1.8) | 1.7 (1.2, 2.4) | 2.5 (1.8, 3.8) | <0.001 |
| HDL-C, mmol/L | 1.4 ± 0.3 | 1.1 ± 0.2 | 1.0 ± 0.2 | 0.9 ± 0.2 | <0.001 |
| LDL-C, mmol/L | 2.6 ± 0.9 | 2.5 ± 0.9 | 2.6 ± 0.9 | 2.5 ± 0.9 | 0.518 |

Values are shown as n (%), mean ± SD, or median (IQR). ALT, AST, TC, TG, and FBG were summarized as median (IQR) and compared across quartiles using the Kruskal-Wallis test. Other continuous variables were compared using one-way analysis of variance. Categorical variables were compared using Pearson chi-square test.

Abbreviations: ALT, alanine aminotransferase; AST, aspartate aminotransferase; BMI, body mass index; FBG, fasting blood glucose; FMR, fat-to-muscle ratio; HDL-C, high-density lipoprotein cholesterol; LDL-C, low-density lipoprotein cholesterol; METS-IR, metabolic score for insulin resistance; MASLD, metabolic dysfunction-associated steatotic liver disease; PLT, platelet count; TC, total cholesterol; TG, triglycerides; UA, uric acid.

**Supplementary Table 2. Association between METS-IR quartiles and MASLD in additional sensitivity models**

| **Model** | **METS-IR quartile** | **n** | **Events** | **OR (95% CI)** | **P value** |
| --- | --- | --- | --- | --- | --- |
| Model 2 + SBP and DBP | Q2 vs Q1 | 235 | 158 | 3.02 (2.04-4.46) | <0.001 |
|  | Q3 vs Q1 | 236 | 176 | 4.01 (2.66-6.03) | <0.001 |
|  | Q4 vs Q1 | 236 | 210 | 9.73 (5.84-16.20) | <0.001 |
| Model 3 + ALT and AST | Q2 vs Q1 | 235 | 158 | 2.67 (1.76-4.04) | <0.001 |
|  | Q3 vs Q1 | 236 | 176 | 3.09 (1.95-4.91) | <0.001 |
|  | Q4 vs Q1 | 236 | 210 | 5.69 (3.15-10.29) | <0.001 |

ORs were estimated for Q2, Q3, and Q4 using Q1 as the reference group. Model 2 was adjusted for age group, sex, tobacco use, alcohol use, and diabetes. Model 3 included Model 2 plus UA, TC, LDL-C, and FMR. Collinearity diagnostics using variance inflation factors did not indicate substantial multicollinearity in the adjusted models. Quartile cut-off values were rounded to two decimal places: Q1 ≤36.78, Q2 >36.78 to <40.98, Q3 ≥40.98 to ≤46.22, and Q4 >46.22.

Abbreviations: ALT, alanine aminotransferase; AST, aspartate aminotransferase; CI, confidence interval; DBP, diastolic blood pressure; FMR, fat-to-muscle ratio; LDL-C, low-density lipoprotein cholesterol; METS-IR, metabolic score for insulin resistance; MASLD, metabolic dysfunction-associated steatotic liver disease; OR, odds ratio; Q, quartile; SBP, systolic blood pressure; TC, total cholesterol; UA, uric acid.

**Supplementary Table 3. Exploratory breakpoint analysis for METS-IR and MASLD**

| **Item** | **n** | **Breakpoint / OR (95% CI)** | **P value** |
| --- | --- | --- | --- |
| Estimated breakpoint | 923 | 38.74 (38.61-38.87) |  |
| METS-IR ≤38.74 | 333 | 1.29 (1.16-1.43) | <0.001 |
| METS-IR >38.74 | 590 | 1.11 (1.06-1.17) | <0.001 |
| Likelihood ratio test | - | - | 0.006 |

The breakpoint analysis was conducted within the 1st-99th percentile range of METS-IR. The effect sizes represent ORs per 1-unit increase in METS-IR below and above the estimated breakpoint. The likelihood ratio test compared the segmented model with a linear model.

Abbreviations: CI, confidence interval; METS-IR, metabolic score for insulin resistance; MASLD, metabolic dysfunction-associated steatotic liver disease; OR, odds ratio.

**Supplementary Table 4. Subgroup analyses for the association between METS-IR and MASLD**

| **Subgroup** | **n** | **Events** | **Adjusted OR per 1-SD increase in METS-IR (95% CI)** | **P value** | **P for interaction** |
| --- | --- | --- | --- | --- | --- |
| Sex |  |  |  |  | 0.046 |
| Female | 203 | 125 | 5.29 (2.91-9.64) | <0.001 |  |
| Male | 740 | 515 | 2.48 (1.97-3.13) | <0.001 |  |
| Age group, years |  |  |  |  | 0.533 |
| 40-49 | 142 | 114 | 2.89 (1.49-5.62) | 0.002 |  |
| 50-59 | 393 | 278 | 3.09 (2.19-4.34) | <0.001 |  |
| 60-69 | 273 | 169 | 2.46 (1.69-3.58) | <0.001 |  |
| 70-79 | 135 | 79 | 3.01 (1.66-5.46) | <0.001 |  |
| Diabetes status |  |  |  |  | 0.220 |
| No diabetes | 564 | 344 | 3.25 (2.45-4.30) | <0.001 |  |
| Diabetes | 379 | 296 | 2.30 (1.64-3.24) | <0.001 |  |

ORs represent the odds of prevalent MASLD per 1-SD (7.30-unit) increase in METS-IR. Sex-stratified models were adjusted for age group, tobacco use, alcohol use, and diabetes. Age-stratified models were adjusted for sex, tobacco use, alcohol use, and diabetes. Diabetes-stratified models were adjusted for age group, sex, tobacco use, and alcohol use. P values for interaction were obtained using likelihood ratio tests comparing models with and without the corresponding multiplicative interaction terms. For categorical age group, the three interaction terms were tested jointly using a 3-degree-of-freedom likelihood ratio test.

Abbreviations: CI, confidence interval; MASLD, metabolic dysfunction-associated steatotic liver disease; METS-IR, metabolic score for insulin resistance; OR, odds ratio; SD, standard deviation.
